# Familial Aggregation of Psoriasis and Co-Aggregation of Autoimmune Diseases in Affected Families

**DOI:** 10.3390/jcm8010115

**Published:** 2019-01-18

**Authors:** Yu-Huei Huang, Chang-Fu Kuo, Lu-Hsiang Huang, Mei-Yun Hsieh

**Affiliations:** 1 Department of Dermatology, Chang Gung Memorial Hospital, Taoyuan 333, Taiwan; huang3764@mail2000.com.tw; 2 Department of Medicine, Chang Gung University, Taoyuan 333, Taiwan; 3 Department of Rheumatology, Allergy and Immunology, Chang Gung Memorial Hospital, Taoyuan 333, Taiwan; shuang1201@gmail.com (L.-H.H.); hsiehmei19@gmail.com (M.-Y.H.)

**Keywords:** psoriasis, familial aggregation, genetic epidemiology, autoimmune diseases

## Abstract

Psoriasis is considered to result from the interaction of genetic factors and environmental exposure. The evidence for familial aggregation in psoriasis has been reported but population-based studies related to the magnitude of genetic contribution to psoriasis are rare. This study aimed to evaluate the relative risks of psoriasis in individuals with affected relatives and to calculate the proportion of genetic, shared, and non-shared environmental factors contributing to psoriasis. The study cohort included 69,828 patients diagnosed with psoriasis enrolled in National health Insurance in 2010. The adjusted relative risks (RR) for individuals with an affected first-degree relative and affected second-degree relative were 5.50 (95% CI (Confidence Interval), 5.19–5.82) and 2.54 (95% CI, 2.08–3.12) respectively. For those who have affected first-degree relatives, their RR was 1.45 (95% CI, 1.17–1.79) for Sjogren’s syndrome and 1.94 (95% CI, 1.15–3.27) for systemic sclerosis. This nationwide study ascertains that family history of psoriasis is a risk factor for psoriasis. Individuals with relatives affected by psoriasis have higher risks of developing some autoimmune diseases.

## 1. Introduction

Psoriasis is a chronic inflammatory cutaneous disorder that results from dysregulated immune reaction mediated by immune cells and keratinocytes [1,2]. The pathogenesis of psoriasis is contributed by genetic susceptibility and environmental stimuli that collectively trigger a cross-interaction between innate and adaptive immune cells [3]. Psoriasis occurs worldwide with prevalence ranging from 0.2% to 3% among different populations [4,5]. In Taiwan, the mean one-year prevalence is 0.23% for men and 0.16% for women respectively [6]. Psoriasis has an impact on the quality of life and was found to be an independent risk factor for cardiovascular diseases, which cause a great disease burden on patients.

Strong evidence has suggested familial clustering in psoriasis. Twin studies reported higher concordance rates for monozygotic twins of 0.20–0.73 compared with 0.09–0.2 for dizygotic twins, and heritability estimates ranging between 66 and 90% [7,8,9,10,11,12]. Epidemiological studies of familial aggregation revealed a high prevalence of psoriasis among relatives and the recurrence risk for affected first-degree relatives were between 4–18.75 [13,14,15]. Although psoriasis is heritable, only part of the disease can be explained by known genetic loci. Currently, evidence from genome-wide association study (GWAS) found that genetic factors only account for less than half of heritability [16]. Environmental factors including stress, infection, trauma to the skin, medication, alcohol, and smoking are considered to participate in the pathogenesis psoriasis [17,18]. However, heritability and contributions of shared environmental factors to phenotypic variance has not been estimated in a population setting. Furthermore, multiple shared genetic loci between psoriasis and other autoimmune diseases have been found in one cross-phenotype meta-anaylsis [19]. Despite these observations, familial co-aggregation of psoriasis and other autoimmune diseases has not been reported in the literature.

This population-based study used a nationwide genealogy reconstruction technique to identify family members of over 20 million people. The individual-level family relationships were linked to health information obtained from Taiwan’s National Health Insurance (NHI) Database, which contains phenotype data regarding health and diseases in essentially the whole population of Taiwan. The aims of this study were to estimate the relative risks (RRs) of psoriasis in individuals with affected relatives and to calculate the proportion of genetic, shared and non-shared environmental factors contributing to psoriasis. In addition, we also examine the RRs of other autoimmune diseases associated with a family history of psoriasis. 

## 2. Material and Methods

### 2.1. Study Population and Data Source

This study was approved by the Institutional Review Board of the Chang Gung Memorial Hospital (104-9794B). We obtained the medical information of study subjects from the Taiwan NHI system, which was established in 1995 and provides health insurance to over 99% of population in Taiwan [20]. The NHI database included extensive information on gender, birthdate, residential location, family relationship, details of insurance, dates of clinical visits and admissions, diagnoses, medical costs, prescriptions, examinations, and procedures. All information for any single individual was connected by a unique personal identification number. All identities were anonymized before the data was released for research to ensure confidentiality. 

### 2.2. Genealogy Reconstruction

The methods of identifying relatives and genealogy reconstruction were described in our previous studies [21,22,23]. The Taiwan NHI system included relationships between beneficiaries, which allowed for a national-level genealogy reconstruction. By the NHI Act and its enforcement rules, parents and grandparents who were unemployed or offspring or grandchildren who were either under 20 years of age and unemployed or over 20 years of age but disable to make a living could serve as dependents of the insured person, an unemployed spouse could serve as dependent. The relationships recorded in the NHI system contained spouse, parent, offspring, and grandparents, grandchildren, great grand-parent and great grand-children on both paternal and maternal sides. Therefore, familial relationships including parent-offspring relationship and spouses could be directly established from the registry by using the indicators of relationships and unique identification numbers. 

The identification of siblings was based on sharing one or more common parents. If a couple did not have offspring, they were not assigned to the same family because they were not related based on a common blood relative. Twins were full siblings who had the same birth date (±1 day) but their zygosity could not be identified from the database.

The registry of beneficiaries was renewed biannually. All the changes including employment, residence, insurance status (insurant or dependent), and relationships between insurant and dependents could incur an individual record in the registry. We used this dynamic nature to maximize the identification of possible family link by incorporating the entire registry records from 1995 to 2010.

We then used the previous links to build pedigrees. We defined a family as a cluster of individuals who were related to each other by blood or by at least one common blood relative. Those subjects without any identified parent were considered founders. We established a pedigree for each founder and then pedigrees were linked if they had common descendants. It should be understood that spouses without a child were not classified into one pedigree. We constructed 4,229,301 families encompassing 21,009,551 parent-child relationships; 17,168,340 full sibling pairs; and 342,066 twin pairs.

### 2.3. Case Definition of Psoriasis and Ascertainment of Other Autoimmune Diseases

The case definition of incident psoriasis was based on dermatologist or rheumatologist diagnosis (International Classification of Diseases, Ninth Revision (ICD-9) codes: 696.1). To ensure the validity, only patients with at least three records in outpatient or inpatient care in 2010 were included in this study. 

We further identified patients with autoimmune diseases by the specific codes from the NHI database including the following: rheumatoid arthritis (RA), systemic lupus erythematosus (SLE), systemic sclerosis, dermatomyositis, polymyositis, Sjögren’s syndrome, multiple sclerosis, and inflammatory bowel diseases.

### 2.4. Covariates

We adjusted covariates that might confound the familial association including age, gender, socioeconomic status (income level, occupation, and residential location), occupation and family size. The income level was calculated approximately based on individual’s insurance premium categories and ranked into gender-specific income quartiles. Occupation is classified into four categories: (1) public servants, teachers, military staff, veterans and their family members; (2) non-manual workers and professionals; (3) manual workers; and (4) others. The residential location for each individual was categorized based on its level of urbanization [24]. 

### 2.5. Statistical Analysis

We defined an individual as a prevalence case when he/she registered in NHI in 2010 and met the case definition of psoriasis between 1 January 1996 and 31 December 2010. The prevalence of psoriasis was estimated for both the general population and individuals with a family history. The relative risks (RRs) of psoriasis calculated in the present study were relative recurrence risks, which were determined as the prevalence of psoriasis among individuals with a specific type of affected relative, divided by the prevalence of psoriasis in the general population [25,26,27]. In addition, RR for spouse was also estimated. The marginal Cox proportional hazards model with an equal follow-up period for all individuals were conducted to measure RRs and 95% confidence intervals (95% CIs) [27]. This model allowed us to estimate adjusted prevalence ratios, which fulfilled the original definition of Riche’s relative recurrence ratios. The RRs were adjusted for age, gender, socioeconomic status and family size and 95% CIs were corrected by the robust sandwich estimator for possible intra-familial clustering. To determine the magnitude of similarity among different types of kinships, tetrachoric correlations for each group of first- and second-degree relatives were calculated by stratification of gender. There was assumption of a continuous normally distributed liability underlying the diagnosis of psoriasis.

Heritability is the proportion of phenotypic variance contributed by genetic factors, while familial additionally considered shared environmental factors. Both measures were calculated using the polygenic liability model [28,29,30,31]. We estimated both the heritability and familial transmission by using the spouse as a control to account for shared environmental factors to phenotypic variance, presuming that spouses shared the family environment without sharing genetic similarity with blood relatives. We confined the family history to first-degree relatives and assumed an average of two sibling members in each family. 

Furthermore, we calculated the degree of familial co-aggregation of other autoimmune diseases in families affected by psoriasis using a marginal Cox proportional hazards regression model with an equal follow-up period for all individuals. RRs and 95% CI for rheumatoid arthritis, systemic lupus erythematosus, systemic sclerosis, dermatomyositis, polymyositis, Sjögren’s syndrome, Behcet’s disease, inflammatory bowel disease, and vasculitis were calculated as adjusted prevalence ratio of specific autoimmune diseases between subjects with a first-degree relative affected by psoriasis and general population. RRs were adjusted for age, gender, family size, and socioeconomic status. Two-tailed *p*-values less than or equal to 0.05 were considered significant. The statistical analysis was performed by SAS v.9.3 (SAS institute, Cary, NC, USA).

## 3. Results

### 3.1. Prevalence of Psoriasis in Individuals with Affected First-Degree and Second-Degree Family Members versus the General Population

**Figure 1:**
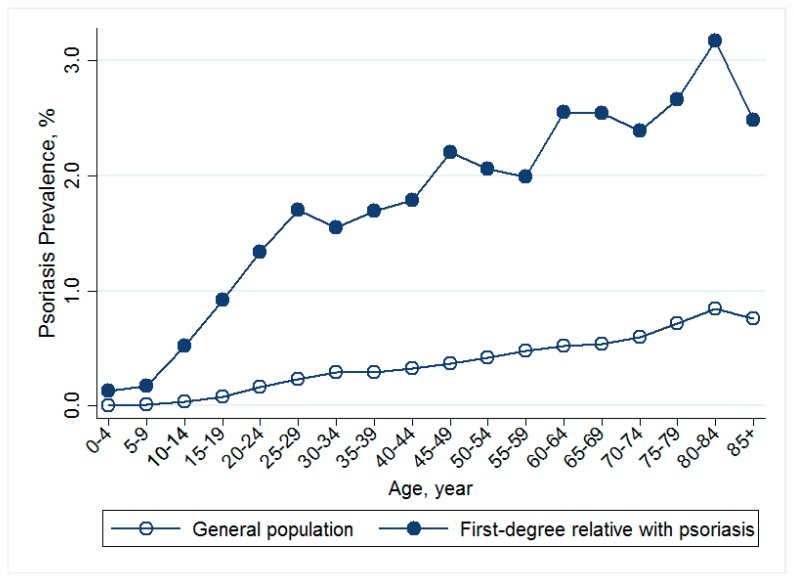
Psoriasis. Age-specific prevalence of psoriasis in individuals with a first-degree relative (circle) with psoriasis and in the general population (hollow circle) in Taiwan in 2010.

The study population included 22,974,772 individuals enrolled in National Health Insurance in Taiwan in 2010. There were 69,828 patients who had a diagnosis of psoriasis in the general population, giving a crude prevalence of 0.30% with a prevalence of 0.22% for women and 0.39% for men (Table 1). In the study population, 140,646 (0.61%) individuals had at least one first-degree relative having psoriasis. Among them, 2198 subjects had psoriasis (prevalence = 1.56%). The numbers of individuals with parent, offspring, siblings and twins affected by psoriasis were 80,100; 32,167; 29,341; and 230 respectively. The age-specific prevalence of psoriasis was higher in individuals with first-degree relatives affected by psoriasis than in the general population (Figure 1).

### 3.2. Relative Risks for Psoriasis in Individuals with Affected First-Degree and Second-Degree Relatives

Prevalence (recurrence risk) of psoriasis in individuals with affected first-degree and second-degree relatives categorized by specific types and gender are shown in Table 2 and Table 3. Overall, the adjusted RRs for individuals with an affected first-degree relative, an affected second-degree relative or an affected spouse were 5.50 (95% CI, 5.19–5.82), 2.54 (95% CI, 2.08–3.12), or 3.63 (95% CI, 3.24–4.07) respectively. Individuals with an affected female first-degree relative were associated with an adjusted RR of 6.55 (95% CI, 6.06–7.07) of having have psoriasis, compared to 5.20 (4.86–5.56) for those with an affected male relative, which suggested gender of the affected relative contributed similarly to RR for psoriasis. 

In comparison with the general population, individuals with one type of affected first-degree relative had a RR of 5.19 (95% CI, 4.9–5.51) and those with two or more had a RR of 27.42 (22.07–34.08) for psoriasis.

The RRs of psoriasis were 63.19 (95% CI, 42.02–95.05) in individuals with an affected co-twin, 7.85 (95% CI, 7.02–8.77) with affected siblings, 5.16 (95% CI, 4.81–5.54) with affected offspring, 5.05 (95% CI, 4.72–5.41) with affected parents, 2.85 (2.17–3.75) with affected grandchildren, 2.63 (95% CI, 1.80–3.86) with one affected niece or nephew, 2.43 (95% CI, 1.90–3.12) with one affected grandparent, 2.19 (95% CI, 1.23–3.99) with one aunt or uncle. 

### 3.3. Familial Transmission and Heritability of Psoriasis

Overall, the tetrachoric correlation coefficients for first-degrees relatives, full siblings and spouse were 0.24 (95% CI, 0.24–0.24), 0.31 (95% CI, 0.30–0.32), and 0.16 (95% CI, 0.16–0.17) respectively (Table 2). The accountability for phenotypic variation of psoriasis was estimated by using a threshold liability model. The proportions of variation due to genetic factors (heritability), shared environmental factors and non-shared environmental factors were 24.5, 30.2, and 45.3% respectively. Based on given parameter, it was estimated that the probability of a psoriasis patient to be sporadic was 83.5%. 

### 3.4. Co-Aggregation of Other Autoimmune Diseases

The relative risks of autoimmune diseases other than psoriasis in individuals with first-degree or second-degree relatives affected with psoriasis compared to general population were shown in Table 4. For those who have affected first-degree relative, their RR was 1.32 (95% CI, 1.16–1.50) for rheumatoid arthritis, 1.45 (95% CI, 1.17–1.79) for Sjögren’s syndrome, 1.25 (1.05–1.48) for systemic lupus erythematosus, and 1.94 (1.15–3.27) for systemic sclerosis. There is no statistically significant increase of RRs in individuals with affected second-degree relatives. 

## 4. Discussion

This is the first nationwide population-based study to investigate the risk of psoriasis and other autoimmune diseases in individuals with affected first-degree or second-degree relatives. We found a 5.5-fold and 2.5-fold increased risk of psoriasis in individuals who have affected first-degree and second-degree relatives than general population, suggesting the association between genetic distance and the risk of psoriasis. Even though familial factors contributed to half of phenotypic variance with one-fourth of total variance attributing to genetic factors, more than 80% of patients were sporadic cases. 

Moreover, individuals with a family history of psoriasis are at increased risk of having RA, Sjögren’s syndrome, SLE, and systemic sclerosis, suggesting common genetic roots shared between psoriasis and these autoimmune diseases. 

Psoriasis is a common, chronic, immune-mediated skin condition, which is currently thought to result from the interaction of genetic factors and environmental exposures [32]. Substantial evidence for family aggregation in psoriasis has been reported but population-based studies related to the magnitude of genetic contribution to psoriasis are scarce. Previous twin studies reported higher concordance rates for monozygotic twins of 0.20–0.73 compared with 0.09–0.2 for dizygotic twins, and heritability estimates ranging between 66 and 90% [7,8,9,10,11,12]. Our estimate derived from the entire population in Taiwan was lower at 24.5%. Epidemiological studies of familial aggregation revealed a high prevalence of psoriasis among relatives and the recurrence risk for affected first-degree relatives were between 4–18.75 [13,14,15]. Our estimate of recurrence risk for affected first-degree relatives was 5.4, which was close to that reported in Swedish study (4) [13]. Compared to the present study, one hospital-based investigation by Di Lernia et al. [14] found much higher recurrence risk for both first-degree (18.75) and second-degree (8.28) relatives. Since psoriasis prevalence has an effect on the measurement of recurrent risk, their assumption of 2% for the prevalence in general population inevitably resulted in higher estimates for the risk.

So far, GWASs have identified 86 susceptibility loci that are associated with psoriasis in European-origin and Chinese populations [33]. However, GWASs only account for 22% and 45.7% of heritability in Europeans and Chinese respectively [16]. The possible explanations for this missing heritability have been proposed including undiscovered rare genetic variants, non-detected gene-gene interaction, lacking of shared environment measurement, heritable epigenetic regulation, and structural variation [33,34,35]. We found significant contribution of shared environment on familial transmission than previous twin studies, implying the extent of missing heritability might be overrated. These results provide helpful information on the risk of heritability and familial transmission for patient education and counseling. Since most of the cases were sporadic, routine screening for the individuals with family members affected by psoriasis does not appear beneficial. 

Our study noted that individuals with a family history of psoriasis had higher risk for psoriatic arthritis, rheumatoid arthritis, Sjogren’s syndrome, systemic lupus erythematosus, and systemic sclerosis and uveitis. Previous evidence for familial co-aggregation between psoriasis and other autoimmune disease is rare. Nevertheless, one nationwide cohort study revealed first-degree relatives of multiple sclerosis patients were at increased risk of psoriasis [36] and multiple shared genetic loci between psoriasis and other autoimmune diseases were revealed in one cross-phenotype meta-analysis [19].

There are some limitations for this present study. First, the definition of cases based on the diagnosis recorded in the registry of NHI database, lacking clinical presentation and severity findings. However, we only included cases diagnosed by experts including dermatologists and rheumatologist; therefore the case definition was rigorous and unlikely to affect the results. Second, we used spouse as a control to estimate heritability, which we might have underestimated due to the effect of assortative mating. However, Heun et al. has found assortative mating can only be of minor relevance in family studies [19]. In addition, we could only consider environmental factors after marriage. Heritability may be overestimated in our study since some familial environmental factors were not evaluated. Third, we could not recognize the zygosity of twins from the database so we could not measure heritability by the classic twin study design. Fourth, we constructed nationwide genealogy based on the family relationships registered in the NHI; thus we could only confirm family relationships for permanent residents in Taiwan. Those who did not depend on family relationships to maintain their eligibility such as Taiwanese citizens living overseas were not included in our study. Furthermore, this study was confined to Taiwan so the application of these results to other populations was uncertain.

In conclusion, this nationwide study ascertains that family history of psoriasis is a risk factor for psoriasis and the effect of environmental factors contributing to this disease was stronger than previously expected. Individuals with relatives affected by psoriasis have higher risk to develop some autoimmune diseases. These results are informative for patient education and counseling as well as future familial studies for psoriasis. 

## Figures and Tables

**Table 1:** Baseline characteristics of individuals with affected first-degree relatives with psoriasis and the general population.

	Women			Men		
Variable	With Affected FDR	General Population	*p*-Value	With Affected FDR	General Population	*p*-Value
No.	69,146	11,651,851		71,500	11,322,921	
Age, mean (SD), *y*	34.6 (18.7)	38.1 (20.6)	<0.0001	34.7 (18.4)	37.5 (20.8)	<0.0001
Psoriasis, no. (%)	971 (1.40)	25,869 (0.22)		1227 (1.72)	43,959 (0.39)	
Place of residence, no. (%)						
Urban	22,713 (32.85)	3,612,878 (31.01)	<0.0001	21,774 (30.45)	3,252,550 (28.73)	<0.0001
Suburban	20,195 (29.21)	3,335,036 (28.62)		20,962 (29.32)	3,191,129 (28.18)	
Rural	26,238 (37.95)	4,703,766 (40.37)		28,763 (40.23)	4,879,055 (43.09)	
Income levels, no. (%)						
Quintile 1	10,495 (15.18)	1,915,419 (16.44)	<0.0001	11,644 (16.29)	2,0616,15 (18.21)	<0.0001
Quintile 2	11,059 (15.99)	1,839,396 (15.79)		9880 (13.82)	1,492,191 (13.18)	
Quintile 3	17,034 (24.63)	3,252,344 (27.91)		18,831 (26.34)	3,218,505 (28.42)	
Quintile 4	15,384 (22.25)	2,297,299 (19.72)		16,380 (22.91)	2,326,478 (20.55)	
Quintile 5	15,128 (21.88)	2,3387,21 (20.07)		14,733 (20.61)	2,217,347 (19.58)	
Occupation, no. (%)						
Dependents of the insured individuals	27,246 (39.4)	4,529,265 (38.87)	<0.0001	24,591 (34.39)	3,781,887 (33.4)	<0.0001
Civil servants, teachers, military personnel, and veterans	2707 (3.910)	414,522 (3.56)		3256 (4.55)	604,575 (5.34)	
Nonmanual workers and professionals	21,502 (31.10)	3,096,472 (26.57)		24198 (33.84)	3,382,170 (29.87)	
Manual workers	12,348 (17.86)	2,699,172 (23.17)		12,308 (17.21)	2,347,070 (20.73)	
Other	5343 (7.73)	912,420 (7.83)		7147 (10.00)	1,207,219 (10.66)	

Abbreviation: FDR, first-degree relative; SD, standard deviation.

**Table 2:** Relative risks of psoriasis in individuals with first-degree relatives diagnosed with psoriasis.

Type of Affected Relative	Gender of Affected Relative	Gender of Individual	No. of Cases	Relative Risks (95% Confidence Interval) ^1^
Any	Male	Male	765	4.81 (4.37–5.29)
		Female	555	5.86 (5.39–6.37)
		All	1320	5.20 (4.86–5.56)
	Female	Male	523	5.41 (4.95–5.90)
		Female	459	8.55 (7.54–9.70)
		All	982	6.55 (6.06–7.07)
	All	Male	1227	4.84 (4.53–5.18)
		Female	971	6.61 (6.14–7.12)
		All	2198	5.50 (5.19–5.82)
Parent	Male	Male	295	4.19 (3.74–4.71)
		Female	183	4.59 (3.98–5.31)
		All	478	4.33 (3.95–4.75)
	Female	Male	235	5.76 (5.05–6.57)
		Female	175	8.69 (7.49–10.09)
		All	410	6.75 (6.10–7.47)
	All	Male	510	4.62 (4.23–5.04)
		Female	349	5.85 (5.27–6.49)
		All	859	5.05 (4.72–5.41)
Offspring	Male	Male	268	4.35 (3.87–4.90)
		Female	237	6.26 (5.52–7.11)
		All	505	5.08 (4.65–5.55)
	Female	Male	161	4.19 (3.60–4.88)
		Female	170	8.14 (7.01–9.45)
		All	331	5.61 (5.04–6.25)
	All	Male	418	4.21 (3.83–4.62)
		Female	396	6.79 (6.15–7.49)
		All	814	5.16 (4.81–5.54)
Sibling	Male	Male	203	7.43 (6.16–8.96)
		Female	146	8.35 (7.09–9.82)
		All	349	7.83 (6.88–8.91)
	Female	Male	139	7.74 (6.55–9.15)
		Female	111	8.8 (6.82–11.36)
		All	250	8.19 (7.06–9.50)
	All	Male	336	7.45 (6.54–8.49)
		Female	251	8.38 (7.26–9.68)
		All	587	7.85 (7.02–8.77)
Twin	Male	Male	18	57.63 (33.87–98.08)
		Female	1	45.7 (7.00–298.5)
		All	19	52.3 (31.37–87.2)
	Female	Male	N/A	N/A
		Female	15	91.95 (48.49–174.38)
		All	15	85.83 (44.27–166.42)
	All	Male	18	51.77 (30.22–88.69)
		Female	16	86.61 (46.94–159.83)
		All	34	63.19 (42.02–95.05)
Spouse	Female	Male	252	3.80 (3.36–4.29)
	Male	Female	263	3.62 (3.21–4.08)
	All	All	515	3.63 (3.24–4.07)

**^1^** Adjusted for age, gender, place of residence, quintiles of income levels, occupation and family size.

**Table 3:** Relative risks of psoriasis in individuals with second-degree relatives diagnosed with psoriasis.

Type of Affected relative	Gender of Affected relative	Gender of Individual	No. of Cases	Relative Risks (95% Confidence Interval) ^1^
Any	Male	Male	42	1.80 (1.21–2.70)
		Female	42	2.40 (1.78–3.24)
		All	84	2.05 (1.58–2.66)
	Female	Male	48	2.94 (2.18–3.95)
		Female	51	4.06 (2.80–5.90)
		All	99	3.42 (2.67–4.38)
	All	Male	86	2.19 (1.70–2.82)
		Female	90	3.03 (2.36–3.89)
		All	176	2.54 (2.08–3.12)
Aunt/uncle	Male	Male	10	1.97 (1.07–3.66)
		Female	7	1.79 (0.85–3.74)
		All	17	1.89 (1.18–3.03)
	Female	Male	1	0.62 (0.09–4.36)
		Female	8	6.25 (2.91–13.43)
		All	9	3.10 (1.51–6.36)
	All	Male	11	1.65 (0.92–2.97)
		Female	15	2.89 (1.69–4.94)
		All	26	2.19 (1.47–3.25)
Niece/nephew	Male	Male	11	2.22 (1.23–3.99)
		Female	1	0.71 (0.10–5.05)
		All	12	1.91 (1.09–3.35)
	Female	Male	8	2.29 (1.15–4.56)
		Female	8	7.96 (4.02–15.77)
		All	16	3.61 (2.16–6.05)
	All	Male	19	2.26 (1.45–3.54)
		Female	9	3.79 (1.99–7.23)
		All	28	2.63 (1.80–3.85)
Grandparent	Male	Male	12	1.45 (0.83–2.55)
		Female	14	2.07 (1.23–3.48)
		All	26	1.72 (1.16–2.56)
	Female	Male	28	3.76 (2.51–5.62)
		Female	19	3.15 (2.01–4.93)
		All	47	3.48 (2.55–4.75)
	All	Male	37	2.37 (1.68–3.34)
		Female	32	2.52 (1.79–3.56)
		All	69	2.43 (1.90–3.12)
Grandchildren	Male	Male	9	1.77 (0.93–3.39)
		Female	20	3.64 (2.36–5.62)
		All	29	2.72 (1.85–4.00)
	Female	Male	11	2.89 (1.61–5.17)
		Female	16	3.75 (2.31–6.10)
		All	27	3.31 (2.26–4.86)
	All	Male	19	2.16 (1.38–3.37)
		Female	34	3.52 (2.52–4.92)
		All	53	2.85 (2.17–3.75)

**^1^** Adjusted for age, gender, place of residence, quintiles of income levels, occupation and family size.

**Table 4:** Relative risks of autoimmune diseases in individuals with affected first- and second-degree relatives affected with psoriasis

Autoimmune Diseases	Gender of Individual	With Affected Relatives		General Population		Relative Risk (95% Confidence Interval) ^1^
		No. of Cases	Prevalence (%)	No. of Cases	Prevalence (%)	
>With affected first-degree relatives with psoriasis						
Rheumatoid arthritis	Male	62	0.09	7739	0.07	1.50 (1.17–1.92)
	Female	175	0.25	29273	0.25	1.27 (1.10–1.47)
	All	237	0.17	37,012	0.16	1.32 (1.16–1.50)
Sjögren’s syndrome	Male	8	0.01	1237	0.01	1.32 (0.66–2.62)
	Female	78	0.11	11,019	0.09	1.47 (1.18–1.84)
	All	86	0.06	12,256	0.05	1.45 (1.17–1.79)
Systemic lupus erythematosus	Male	13	0.02	1963	0.02	1.06 (0.61–1.82)
	Female	124	0.18	16,646	0.14	1.27 (1.07–1.52)
	All	137	0.10	18,609	0.08	1.25 (1.05–1.48)
Systemic sclerosis	Male	1	0.001	377	0.003	0.47 (0.07–3.29)
	Female	14	0.02	1451	0.01	1.45 (1.17–1.79)
	All	15	0.01	1828	0.01	1.94 (1.15–3.27)
Polymyositis/Dermatomyositis	Male	6	0.01	529	0.004	1.86 (0.84–4.13)
	Female	3	0.004	1219	0.01	0.46 (0.15–1.43)
	All	194	0.14	34,000	0.15	1.17(1.01–1.35)
Inflammatory Bowel Disease	Male	16	0.02	1651	0.01	1.55 (0.95–2.52)
	Female	4	0.01	993	0.01	0.76 (0.29–2.04)
	All	20	0.01	2644	0.01	1.29 (0.83–1.99)
Multiple Sclerosis	Male	1	0.001	279	0.002	0.53 (0.07–3.73)
	Female	7	0.01	926	0.01	1.29 (0.62–2.71)
	All	8	0.01	1205	0.01	1.09 (0.55–2.19)
With affected second-degree relatives with psoriasis						
Rheumatoid arthritis	Male	6	0.02	7739	0.07	0.89 (0.40–1.97)
	Female	24	0.10	29273	0.25	1.13 (0.76–1.69)
	All	30	0.06	37,012	0.16	1.07 (0.75–1.52)
Sjögren’s syndrome	Male	2	0.01	1237	0.01	2.38 (0.60–9.54)
	Female	9	0.04	11,019	0.09	1.23 (0.60–2.52)
	All	11	0.02	12,256	0.05	1.30 (0.69–2.47)
Systemic lupus erythematosus	Male	4	0.02	1963	0.02	1.78 (0.67–4.76)
	Female	16	0.07	16,646	0.14	0.99 (0.61–1.62)
	All	20	0.04	18,609	0.08	1.09 (0.70–1.68)
Systemic sclerosis	Male	N/A	N/A	377	0.003	N/A ^2^
	Female	N/A	N/A	1451	0.01	N/A
	All	N/A	N/A	1828	0.01	N/A
Polymyositis/Dermatomyositis	Male	1	0.004	529	0.004	1.62 (0.23–11.57)
	Female	1	0.004	1219	0.01	0.87 (0.12–6.24)
	All	32	0.07	34000	0.15	1.17(0.82–1.67)
Inflammatory Bowel Disease	Male	2	0.01	1651	0.01	1.25 (0.31–4.99)
	Female	N/A	N/A	993	0.01	N/A
	All	2	0.004	2644	0.01	0.81 (0.20–3.22)
Multiple Sclerosis	Male	N/A	N/A	279	0.002	N/A
	Female	1	0.004	926	0.01	1.18 (0.17–8.36)
	All	1	0.002	1205	0.01	0.84 (0.12–5.96)

**^1^** Adjusted for age, gender, place of residence, quintiles of income levels, occupation and family size. **^2^** Not applicable (N/A) because of no cases with affected second-degree relatives with psoriasis.
